# Flexural Strength Enhancement of Lightweight Concrete Beams Using Carbon Fiber and Epoxy Resin

**DOI:** 10.3390/ma18235297

**Published:** 2025-11-24

**Authors:** Damian Markuszewski, Paweł Ciężkowski, Ilmars Blumbergs

**Affiliations:** 1 Faculty of Automotive and Construction Machinery Engineering, Division of Machine Design Fundamentals, Warsaw University of Technology, Ludwika Narbutta 84 Street, 02-524 Warsaw, Poland; damian.markuszewski@pw.edu.pl; 2 Faculty of Automotive and Construction Machinery Engineering, Division of Numerical Methods and Intelligent Structures, Warsaw University of Technology, Ludwika Narbutta 84 Street, 02-524 Warsaw, Poland; 3 Faculty of Engineering and Management, Riga Aeronautical Institute, 9 Mezkalna Street, LV-1058 Riga, Latvia; i.blumbergs@rai.lv

**Keywords:** aerated concrete, concrete strength, volcanic rock, carbon fiber-reinforced concrete, bending behavior

## Abstract

The paper presents the effect of reinforcing aerated concrete and volcanic rock on flexural strength. The tests used a three-point flexural test on beams made of both materials and two types of reinforcement: an Epidian 5 epoxy resin coating and carbon fabric impregnated with the same resin. The carbon composite increased the strength of aerated concrete by 1940% and volcanic rock by 2633% compared to unreinforced samples. The resin alone improved the load-bearing capacity of the beams by aerated concrete 118% and volcanic rock 147%, respectively. The tests showed that the resin filled the pores of the porous materials, significantly increasing their flexural strength.

## 1. Introduction

Concrete can be produced in various variants and adapted to specific applications. Examples of concretes used in construction: self-compacting concrete, which flows into the formwork without vibration [1,2,3,4,5], concrete with dispersed reinforcement, increasing resistance to cracking [6,7,8], self-leveling concrete for flooring applications [9,10,11], lightweight concrete [12,13,14,15], high-strength concrete [16,17,18], architectural concrete [19,20,21,22,23,24], and waterproof and abrasion-resistant concrete [25,26].

In recent years, increasing attention has been paid to the use and development of energy-efficient building insulation materials [27,28,29,30]. These requirements are met by aerated concrete, which is characterised by low density, can have solid waste added during production, and has fire resistance, thermal insulation, and sound absorption properties [31], thanks to which it has found wide application in construction [32,33,34]. Obtaining insulation materials used in construction with high strength and low density is the goal and direction of research pursued by scientists.

Aerated concrete is a type of lightweight concrete made from cement or lime mortar, in which air voids are introduced into the matrix using an air-entraining agent [35,36,37,38,39]. It is typically produced from sand or sand mixed with fly ash as aggregate, with lime and a small amount of cement as the binder. Aluminum powder or paste acts as a foaming agent, reacting with sodium hydroxide to release hydrogen, which forms the characteristic pores in the material.

The main advantages of lightweight concrete include low density, which reduces structural loads and transport costs; ease of processing, as elements can be easily cut or shaped; high fire resistance; and good thermal insulation resulting from its porous structure, beneficial for energy-efficient buildings [40,41,42]. It is also noted for its durability.

Volcanic rock is a natural, porous, and lightweight aggregate formed by the rapid cooling and solidification of molten lava. After appropriate preparation, it can be used as an aggregate for the production of lightweight concrete or as a cementitious component in the manufacture of concrete or geopolymers [30,43]. Volcanic rock, also known as pumice, is an easily accessible, environmentally friendly, economical, and lightweight building material. In the cosmetics industry, it is marketed under the trade name pumice.

The compressive strength of aerated concrete is usually in the range of 1.7–4.0 MPa. Most of the methods proposed in the literature [44] for improving the strength of lightweight concrete involve modifying the composition of the mixture by adding, for example, fibers [45], using graphite tailings as an alternative silica source [46], using polypropylene fibers of various lengths [45], using red gypsum [47], steel slag, and many other materials [44]. The most advantageous method of reinforcement was proposed in [47], which achieved an increase of 1100 kg/m^3^, and the maximum compressive strength value was 10.38 MPa.

In order to increase the flexural strength of lightweight concrete, fiber additives are used, but their introduction is associated with a reduction in the workability of the concrete mix [48,49,50,51].

Textile-reinforced concrete (TRC) has high tensile strength and high durability, with the potential for constructing thin, modular structures with low weight and slender elements. This composite is increasingly used in engineering applications [52,53,54,55,56]. The literature also describes many applications of glass fibre-reinforced polymer (GFRP) rods (FRP) rods as an external reinforcement for reinforced concrete structures has gained popularity due to the favourable properties of FRP, such as high strength-to-weight ratio, ease and speed of application, corrosion resistance and minimal change in the geometry of structural elements [57,58].

Concrete, as a basic construction product, is exposed to destructive environmental influences [59,60,61,62], resulting in the degradation of its structure, which in turn affects its performance properties. Examples of destructive processes are described in the PN-EN 1992-1-1:2024-05 standard [63]. Currently, the primary goal of research on the durability of concrete is to improve its properties, which allows the durability of constructed buildings to be maintained at the required level. One of the methods of protecting concrete is the use of special plasters, protective coatings [64,65], and paints that prevent aggressive factors from penetrating deep into the concrete structure. There are studies available in the literature showing the beneficial effect of protecting concrete with a coating of Epidian-5 resin and Z−1 hardener (Impregnation with Epidian−5 resin and Z−1 hardener [66,67]), which resulted in a 65% improvement in the flexural strength of concrete compared to unprotected samples [68]. In the studies presented in the paper, the strength of the concrete decreased with the increase in the time it spent in an aggressive environment [69].

Two methods of reinforcing the test specimens were used in this study. The first method involved coating the samples with a two-component epoxy resin—Epidian 5. The second method involved reinforcing the samples by applying carbon fabric, attached to the beams using the same resin, in order to improve their flexural strength [70,71,72]. Both methods contributed to varying degrees to increasing the strength of the test samples. There is a lack of studies in the current literature documenting the use of carbon fabrics to reinforce lightweight concrete, which justifies the need to conduct experiments to verify the feasibility of implementing the proposed reinforcement method in industrial applications. This study is expected to provide important baseline data for future industrial implementations [73].

## 2. Materials and Methods

### 2.1. Materials

For the study, aerated concrete (AR) of class 600, i.e., with a density of 600 kg/m^3^, was used, and this decision was made due to its widespread use. According to the manufacturer’s data, this concrete complies with the EN 771-4:2011+A1:2015 standard [74]. The second material used in the experiment was volcanic rock (VR). The experiment used Epidin 5 epoxy resin, which is characterized by: versatility in combining materials with different properties, impregnation of various materials, minimal shrinkage during curing, and the possibility of curing at room temperature. For the experiment, Epidian 5 epoxy resin and Z1 hardener were used in a ratio of 200 g of resin to 24 g of hardener. The components were mixed manually with a wooden stirrer for 3 min. The prepared resin was used to impregnate a carbon fibre mat, and the excess was squeezed out with a boatbuilding roller for 30 min until the mixture gelled at 22 °C and a humidity of 47.7%. The tests were performed after 48 h, when the composite had probably reached a strength of 80–90%. It is likely that if the experiment had been carried out after 14 days, the strength of the sample could have increased further. Carbon fabric weighing 200 g/cm^2^ and a 1/1 twill weave was used as the reinforcing fabric. One layer of reinforcing composite is 0.2 mm thick, and its orientation relative to the longest dimension is 0/90/0.

In the case of sample AC3, the impregnated carbon fibre mat was pressed with a boatbuilding roller until the resin gelled. In the case of sample AC6, the resin-impregnated fabric was pressed with a boatbuilding roller, then wrapped in release film and pressed with non-stretch tape. This method of sample preparation significantly improved the strength parameters. A carbon mat with a plain weave (1/1) is characterised by high stability, stiffness and resistance to slippage, which makes it ideal for simple elements and reduces resin consumption. Its disadvantage is lower flexibility and less susceptibility to forming compared to a twill weave, especially in the case of complex shapes. The aerated concrete and carbon mat were purchased online. Aerated concrete—MIAMI Tomasz Zawadzki Sp. z o.o., Kasztanowa 6, 76-200 Słupsk, carbon mat—https://www.tkane-composites.pl/ (accessed on 10 November 2025). The volcanic rock was purchased in Turkey in the city of Sanliurfa—Fe Topuk Taşi pumice stone 8693203014190.

### 2.2. Specimens’ Preparation

**Figure 1:**
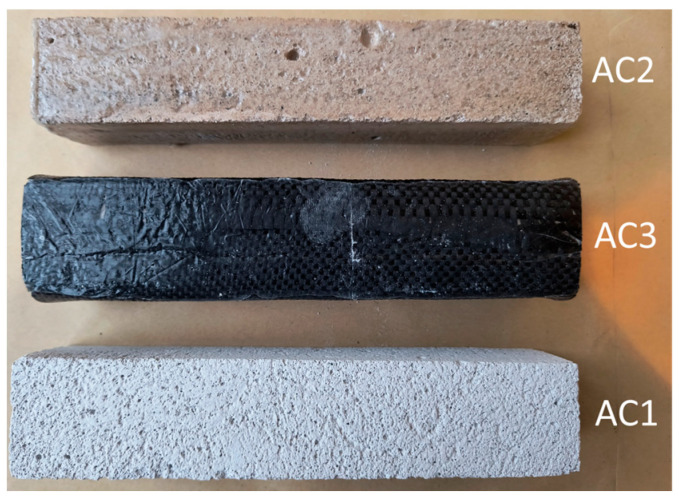
Prepared samples of aerated concrete.

**Figure 2:**
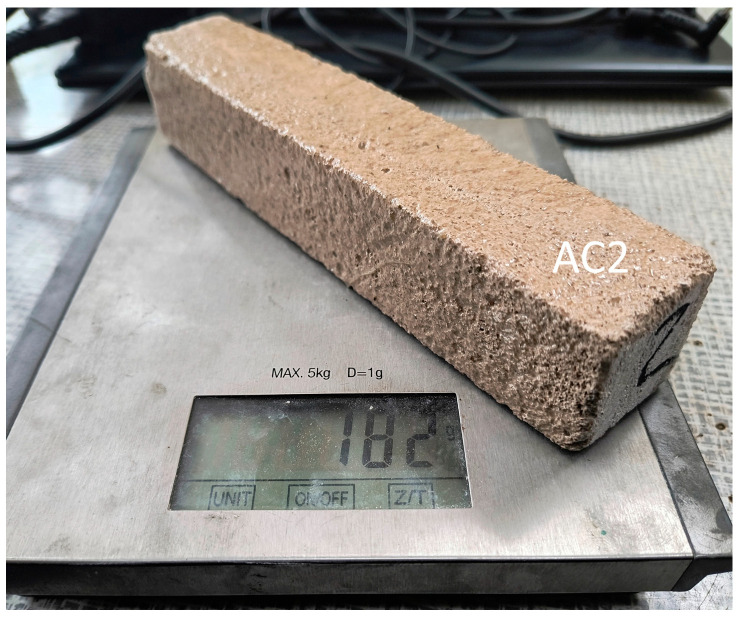
Determination of sample mass.

Figure 1 and Figure 2 show the prepared samples of aerated concrete for testing.

Preliminary tests of the flexural strength of aerated concrete were carried out on samples measuring 40 × 40 × 200 mm (Figure 1).

Next, in order to check the impact of the resin coating on the strength of the materials, the samples were impregnated—the first sample without additives (AC1), the second sample was impregnated with resin (AC2), and the third sample was additionally reinforced with carbon fabric (AC3). The test was carried out four days after the preparation of the samples—Table 1.

The purpose of the resin was to infiltrate the capillary pore system, fill it, and seal it. This process aimed to improve the material’s structural integrity and increase its mechanical strength. Bending strength tests were performed on individual samples, which allowed the change in strength of protected and unprotected samples to be determined.

Samples of aerated concrete and volcanic rock measuring 20 × 20 × 110 mm were prepared for the main experiment. The new sample dimensions were related to the change in the possibility of purchasing a base plate of volcanic rock measuring 20 × 70 × 110 mm, from which three samples were made. The new aerated concrete samples had the following weights: sample without additives (AC4), sample impregnated with resin (AC5), and sample additionally reinforced with carbon fabric (AC6). The masses of the volcanic rock samples were as follows: sample without additives (VR1), sample impregnated with resin (VR2), and sample additionally reinforced with carbon fabric (VR3)—Table 1. The accuracy of the weight measurement is +/−1 g.

### 2.3. Flexural Strength Test

**Figure 3:**
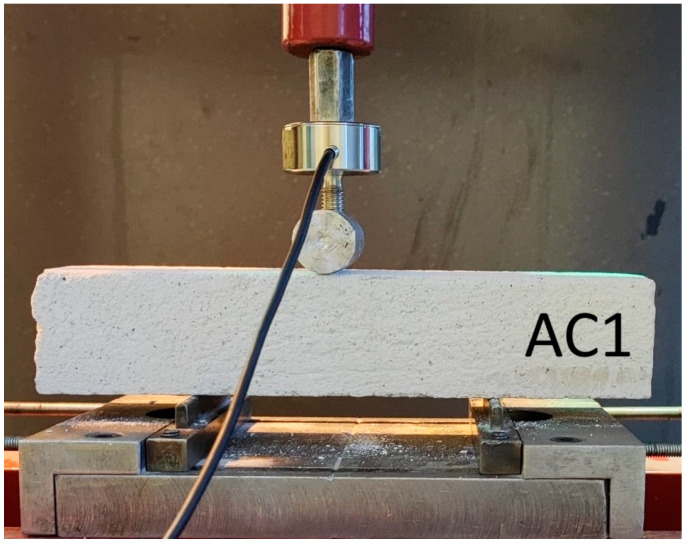
Preliminary experiment.

**Figure 4:**
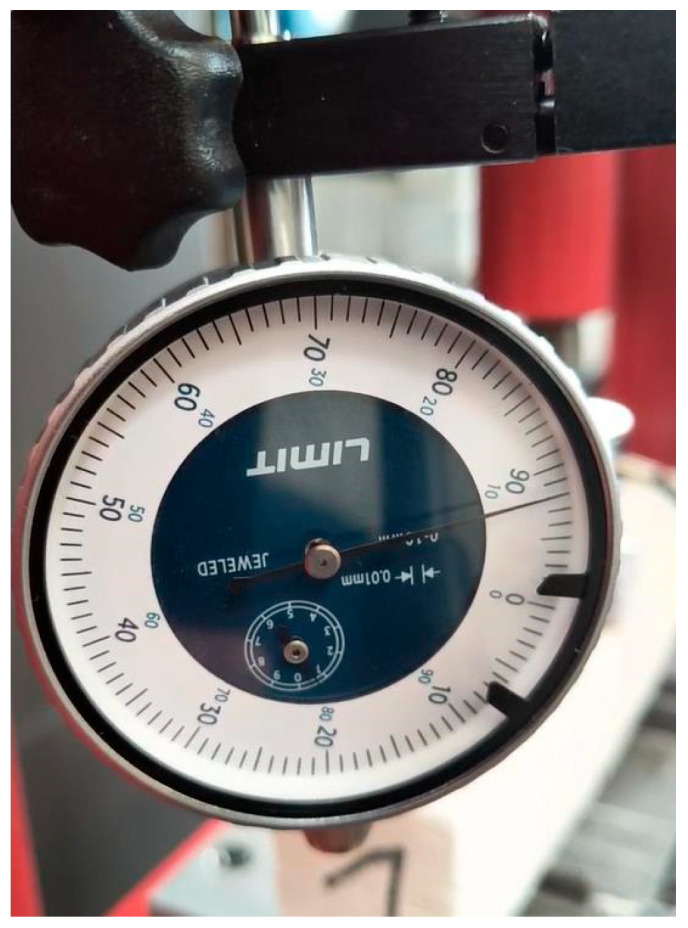
Beam deflection measurement.

**Figure 5:**
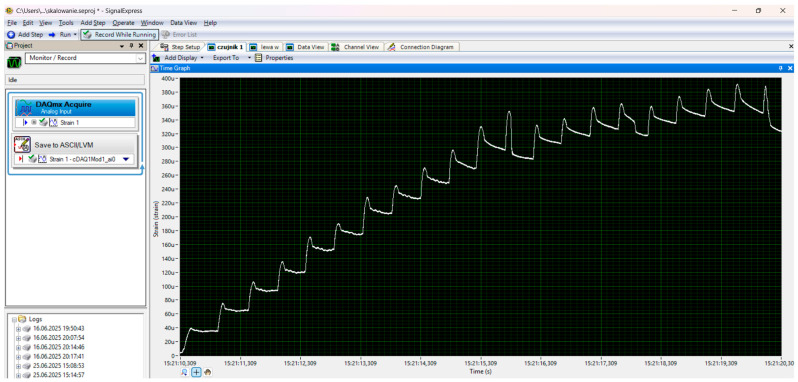
Recording program.

To estimate the maximum destructive force, a preliminary experiment was carried out on a hydraulic press with a cylinder pressure of 12 tons (Figure 3). During the tests, the maximum force and deflection of the beam were recorded using a dial gauge (Figure 4). The distance between the beam supports was set at 100 mm. The signals were recorded using a Wobit KMM20 force sensor (WObit, Pniewy, Poland) linearity tolerance 0.25%, an eDAQ-9178 chassis (National Instruments, Austin, TX, USA) for measurement systems with a multi-channel module equipped with NI 9234, and LabView Signal Express 2015 software on a laptop computer (Figure 5).

**Figure 6:**
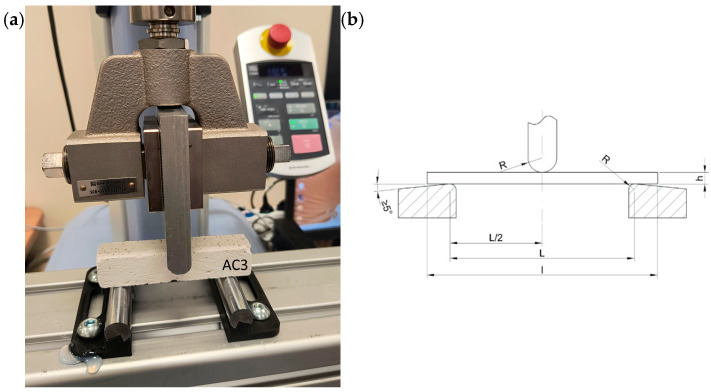
(**a**) Fundamental experiment—flexural strength test applied to test specimen, (**b**) three-point bending test (h—sample thickness, l—sample length, L—support spacing, R—support or bending mandrel radius), ISO 178:2019 [75].

The main experiment was performed on a single-column tensile testing machine manufactured by Shimadzu (Kyoto, Japan), model EZ-test-LX (Figure 6a). The device is designed for composite materials, among other things. The measurements were recorded at 100 Hz for a head with a measuring range from 0 N to 5000 N and a test speed range of 1 mm/min and linearity tolerance 0.5%. The recorded data was saved on a computer. The data was analysed using MATLAB 2024b software. The distance between the beam supports was 80 mm.

The data analysis was performed per the guidelines in ISO 178:2019 [75] and EN 196-1:2016 [76]. Both standards concern triaxial bending tests (Figure 6b) of composite and concrete materials. Based on the tests and the results obtained, the flexural strength $\;\delta_{fM}$ and flexural strain $\varepsilon_{fM}\;$ were determined using the following formulas:

(1) $$\delta_{fM}=\frac{3\cdot F_{M}\cdot L}{2\cdot b\cdot h^{2}}$$

where

$F_{M}$—maximum recorded force in N;
L—distance between supports in mm;
b—sample width in mm;
h—sample thickness in mm.

(2) $$\varepsilon_{fM}=\frac{600\cdot s\cdot h}{L^{2}}$$

where

L—distance between supports in mm;
s—deflection at a given point in mm;
h—sample thickness in mm.

### 2.4. Microscopic Observations

The microstructure was observed using a Leica M205A stereoscopic microscope (Leica, Wetzlar, Germany) [77]. A 20 × 20 mm sample break was obtained from the manufactured samples (remaining parts after the bending test). Observations were carried out using magnifications of up to ×11.7.

## 3. Results

### 3.1. Preliminary Experiment

In the first recognition experiment, the destruction strength and maximum deflection were determined for AC1, AC2, and AC3 samples. The results obtained are presented in Table 2.

**Figure 7:**
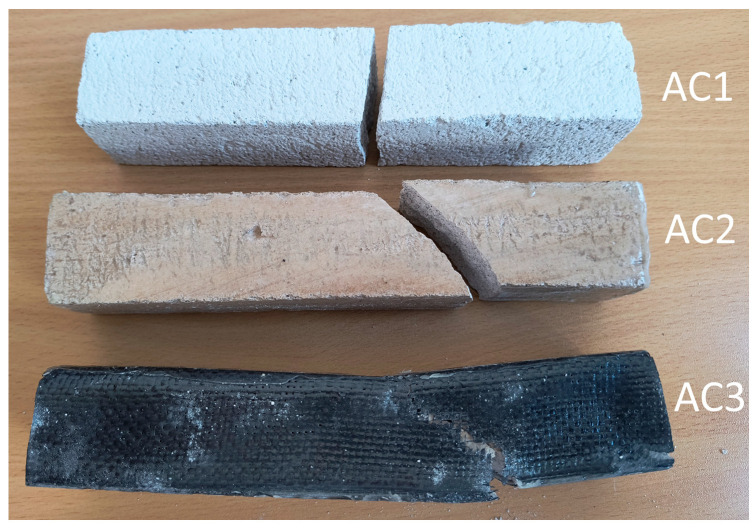
Crack patterns of flexural beams at failure. The samples examined 40 × 40 × 200 mm: AC1, AC2, AC3.

**Figure 8:**
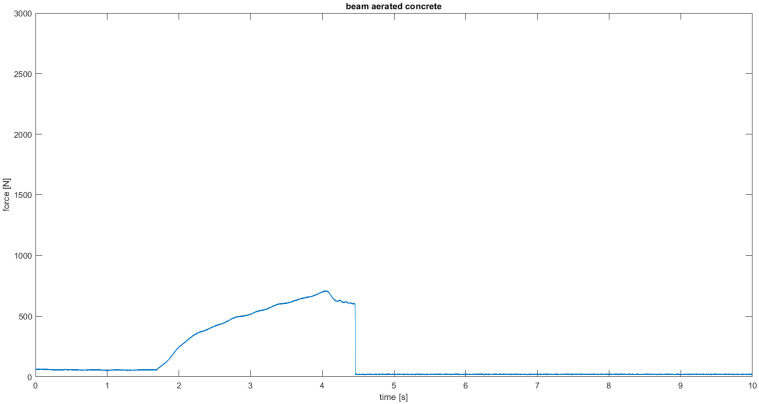
Load–time relationship of flexural beams for AC1.

Figure 7 shows the shape and pattern of cracks in the tested samples. The highest flexural strength was obtained for the AC3 sample reinforced with carbon fiber, reaching 361% of the base value for the base aerated concrete. Adding resin alone resulted in a 148% increase in strength compared to the base value for the base aerated concrete. The fracture mechanism can be seen at the breaks in the samples (Figure 7). The aerated concrete sample fractured exactly where it was expected to, i.e., in the middle of the beam, where the deflection reached its maximum value and, consequently, the bending stresses (Figure 8). The reinforcing beams’ cracking was initiated by the support, where the permissible surface pressure stresses were probably exceeded. The bending strength graph for the carbon composite beam shows a sudden drop in strength in two places caused by the crushing of the internal aerated concrete core. The reinforcement in the form of the composite is so strong that it can continue to transfer the load despite the damage to the core up to the maximum value.

### 3.2. Fundamental Experiment

Due to the imperfections of the experiment in the form of a sudden increase in force resulting from the use of a manual hydraulic pump drive (Figure 5), the authors decided to conduct the main experiment on a Shimadzu EZ-test-LX strength testing machine at a constant speed range of 1 mm/min. The results obtained are presented in Table 3. The main test compared the flexural strength of aerated concrete and volcanic rock samples with their reinforcements.

**Figure 9:**
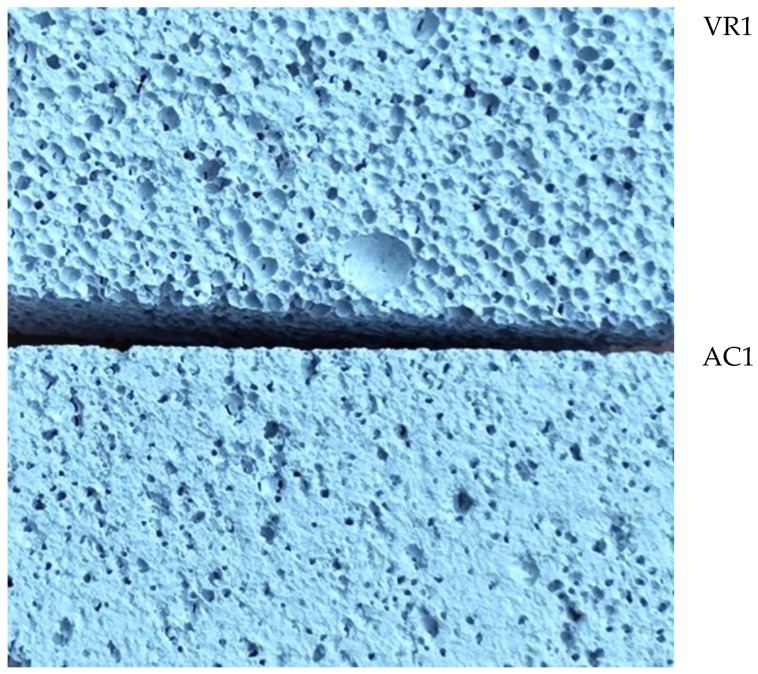
Surface area of VR1 volcanic rock and AC1 aerated concrete samples not reinforced prior to destruction.

Figure 9 shows the surface of aerated concrete and unreinforced volcanic rock samples before destruction. There are clear differences in the structure of the materials—volcanic rock has a porous structure, while aerated concrete has a more compact structure with fewer visible pores.

**Figure 10:**
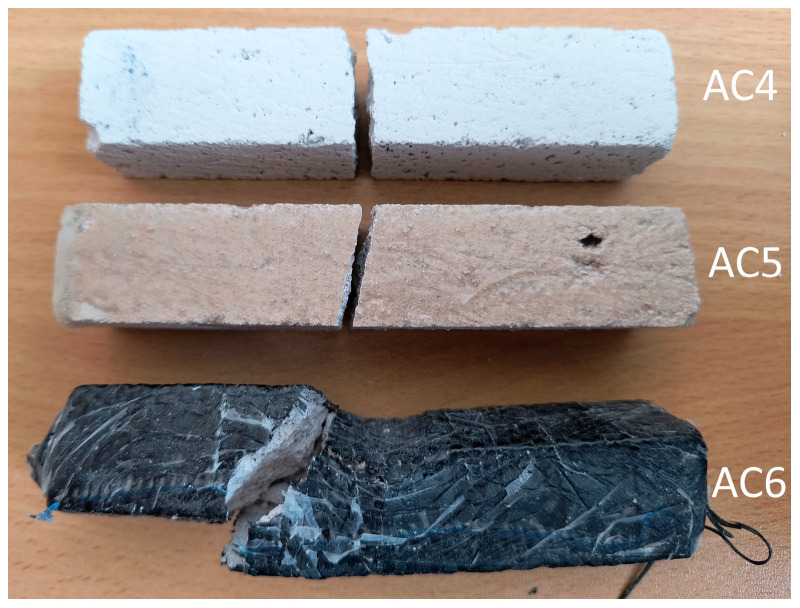
Crack patterns of flexural beams at failure. The samples examined 20 × 20 × 110 mm: AC4, AC5, AC6.

**Figure 11:**
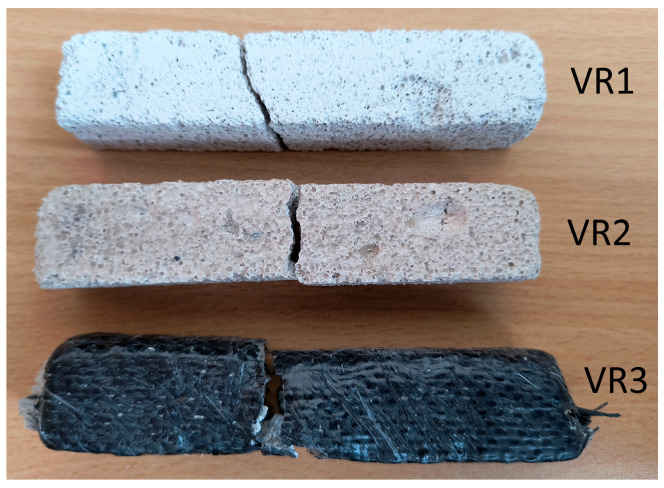
Crack patterns of flexural beams at failure. The samples examined 20 × 20 × 110 mm: VR1, VR2, VR3.

Figure 10 and Figure 11 show the fracture surfaces of the samples from the basic experiment.

**Figure 12:**
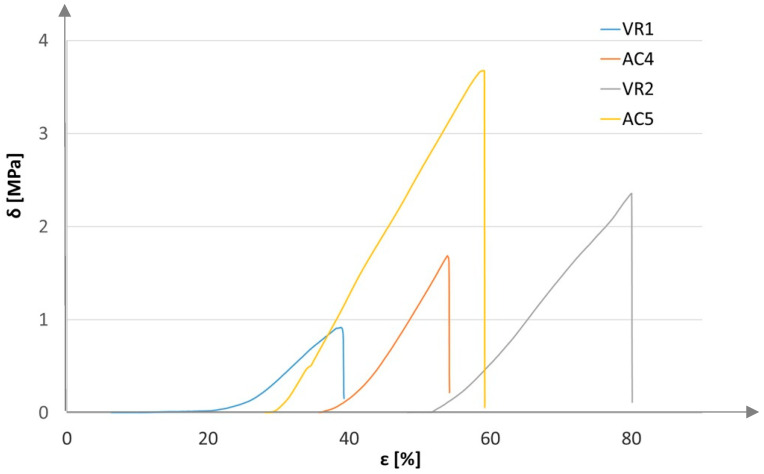
Bending strength characteristics for base and reinforced samples.

**Figure 13:**
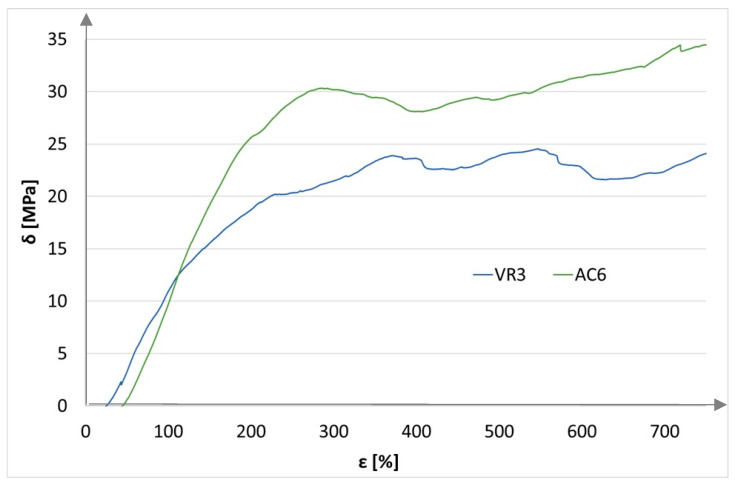
Bending strength characteristics of samples reinforced with a single layer of carbon mate.

For the recorded measurement values, bending strength characteristics were plotted: stress as a function of strain (Figure 12 and Figure 13).

Figure 12 shows increased flexural strength for samples reinforced with a resin layer of aerated concrete and volcanic rock. The only difference is the duration of bending. The base sample of unreinforced volcanic rock was locally crushed, and the bending process was delayed. In contrast, the bending of the other samples proceeded according to the procedure. The starting point for all characteristics was the tensometric force sensor (strain gauge) reading from a value of 0.1 N.

Figure 13 shows a significant increase in the strength of samples reinforced with resin and a single layer of carbon fabric.

**Figure 14:**
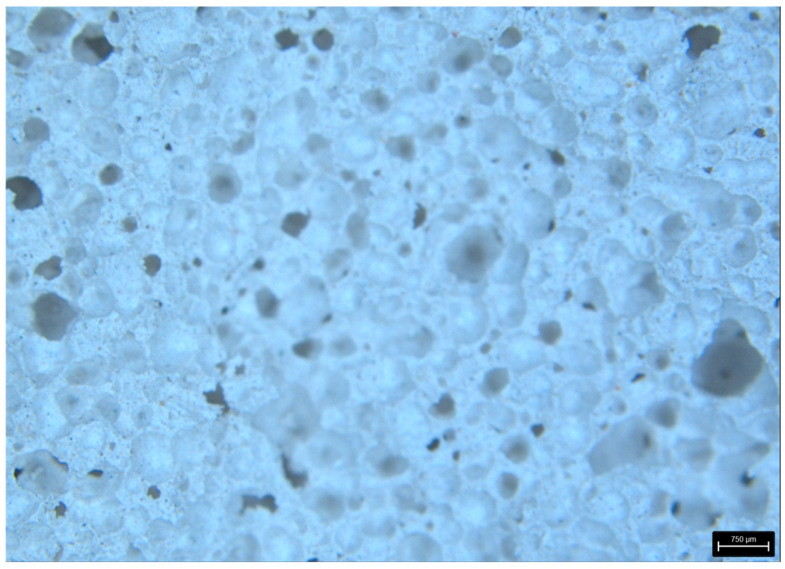
AR—aerated concentrate—surface area of a break.

**Figure 15:**
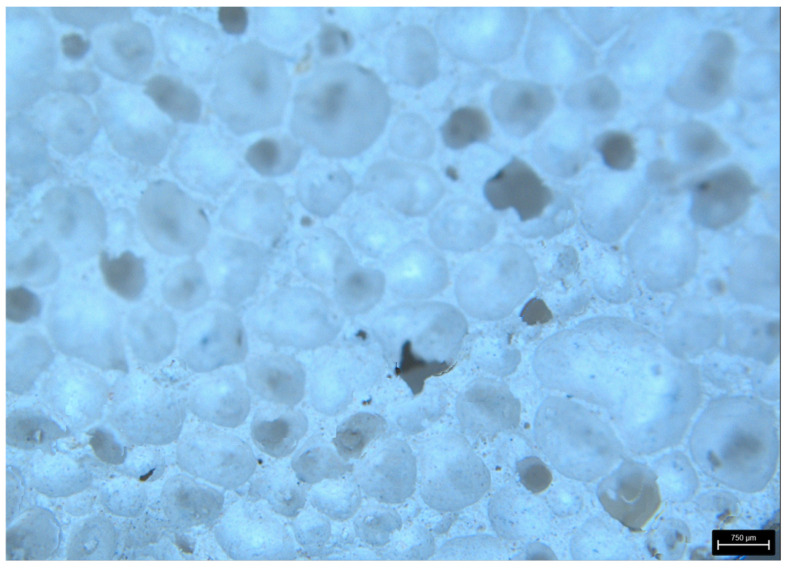
VR—volcanic rock—surface area of a break.

In addition, photographs of fractures for samples without reinforcement are presented (Figure 14 and Figure 15).

As can be observed in the structure of aerated concrete, the air bubbles are much smaller than in volcanic rock. This is also reflected in the weight of the samples, where the weight of the aerated concrete sample is 55% greater than that of the volcanic rock.

## 4. Discussion

The authors conducted a literature review and found two publications to compare their results. The first publication is [78]. This publication presents research on the strength parameters of aerated concrete. During the experiment, in a three-point bending test of aerated concrete samples of class 600, a bending strength of 1.14 MPa was obtained, and for class 700—1.83 MPa. The authors of this article obtained 1.84 MPa for Ytong PP4/06 aerated concrete and class 600 declared by the manufacturer.

The second publication on this subject is [68]. During the experiment, the effect of protection in the form of Epidian 5 epoxy resin and water glass on the flexural strength of concrete was tested. During the flexural strength tests, the following results were obtained: base concrete flexural strength 3.62 MPa, concrete protected with water glass 3.98 MPa—10% increase in strength, and concrete protected with epoxy resin 5.96 MPa—65% increase in strength. During the main experiment, the authors of this article obtained the following results for the base aerated concrete 1.69 MPa, 3.68 MPa for aerated concrete coated with epoxy resin—a 118% increase in strength, and 34.47 MPa for aerated concrete coated with epoxy resin and a single layer of carbon fabric—a 1940% increase in strength. For volcanic rock, the following values were obtained for the base sample: 0.92 MPa, volcanic rock coated with epoxy resin 2.27 MPa—strength increase of 147%, volcanic rock coated with epoxy resin and one layer of carbon fabric 25.14 MPa—strength increase of 2633%.

**Figure 16:**
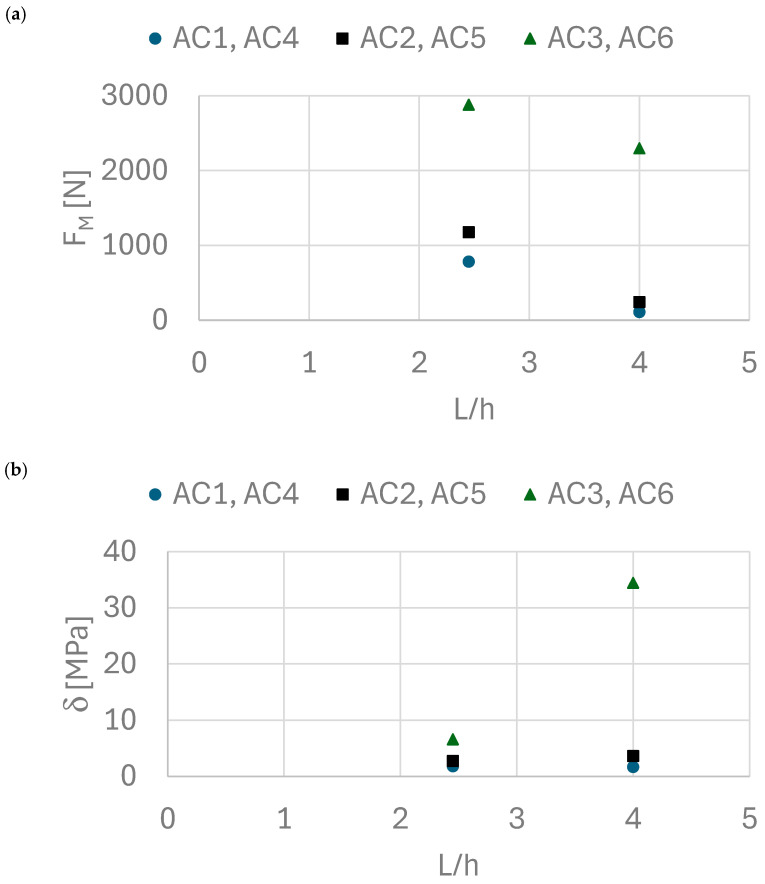
(**a**) Dependence of force on the L/h ratio, (**b**) dependence of bending stress as a function of the L/h ratio.

Figure 16 compares the experimental results of force values and bending stress values for aerated concrete. The results show that the force values differ slightly, while the stress values for unreinforced and resin-reinforced samples are similar. However, an increase in strength and bending was observed when the carbon mat application technique was changed.

The results obtained in the preliminary experiment and the main experiment are consistent, except for the results for reinforcement with a single layer of carbon fiber mat. In the main experiment, manual lamination was performed until the composite was cured. In contrast, the main experiment used polyester tape to press the carbon fiber mat against the core. This technological measure resulted in better adhesion of the carbon fiber mat to the core and the squeezing out of excess resin. This translated into a significant increase in flexural strength from 6.66 MPa to 34.47 MPa.

Due to limited access to volcanic rock material, they restricted their research to one sample of each type: a sample without additives, a sample with resin additives, and a sample with a layer of carbon fibre.

**Figure 17:**
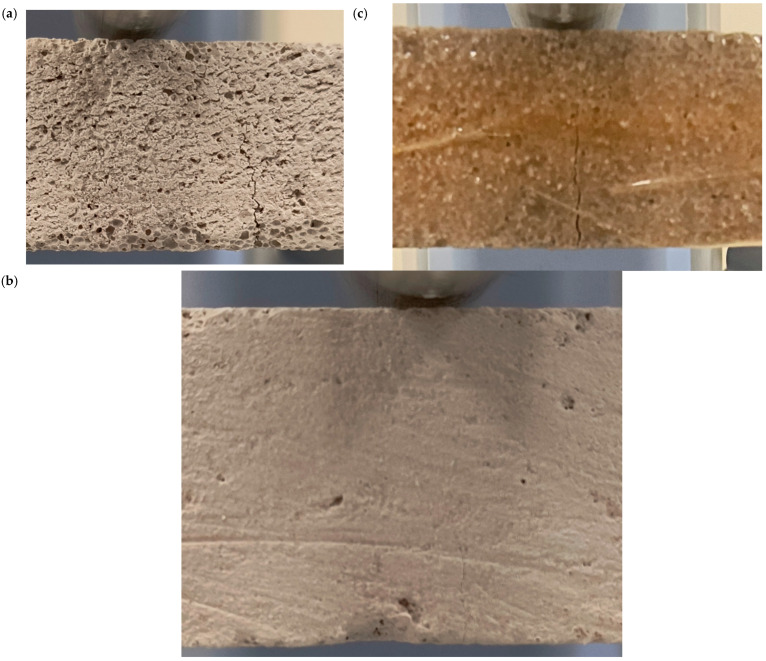
(**a**) Fracture pattern of sample VR1, front view, (**b**) fracture pattern of sample AC5, front view, (**c**) fracture pattern of sample AC4, front view.

The failure of a brittle material stems primarily from the loss of cohesion within its structure. In practice, this means that microscopic flaws—such as tiny pores or cracks—begin to expand rapidly under increasing load. These cracks can cut through grain boundaries or other weak spots in the microstructure, which quickly leads to the complete destruction of the component. What stands out is that a brittle material breaks apart with virtually no visible plastic deformation, and I have to admit that this sudden, violent nature of the process always commands a certain respect: it makes predicting the exact moment of failure remarkably difficult. Figure 17 shows the cracks observed in selected samples as a result of the pressure exerted by the upper punch on the bent sample.

**Figure 18:**
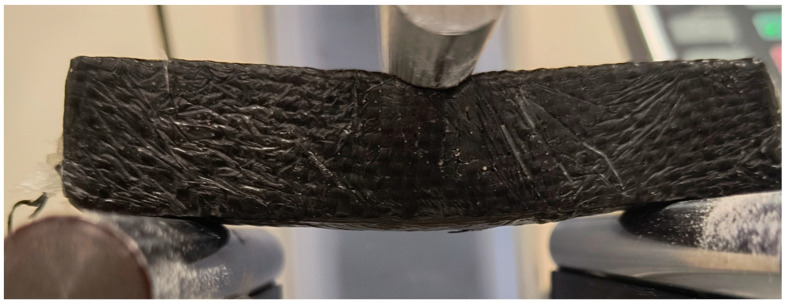
Sample VR3 reinforced with carbon mat during three-point bending test.

In the case of a sample reinforced with a single layer of carbon fibre mat, the mechanism of destruction of the bent sample occurs through the deflection and fracture of the outer cladding layer and the cracking of the core [79] (Figure 18).

The occurrence of local stresses and deformations, sudden changes in the shape of the core structure, as well as manufacturing imperfections make it impossible to assess strength at the macrostructural level [80].

## 5. Conclusions

The study’s main objective was to determine the effectiveness of resin and carbon fabric in reinforcing lightweight concrete beams and volcanic rock beams during a three-point bending test. As expected, the reinforced beams showed a significant increase in bending strength compared to the unreinforced beams. Thanks to the use of resin reinforcement layers in the first case and resin and carbon fabric layers in the second case, it was possible to achieve an increase in bending load capacity of 118% (lightweight concrete) and 147% (volcanic rock) in the first case, and 1940% (lightweight concrete) and 2633% (volcanic rock) in the second case.

The significant increase in flexural strength is due to a change in the sample manufacturing technology. In the case of the AC3 sample, the impregnated carbon fibre mat was pressed with a roller used in boat building until the resin gelled. In the case of the AC6 sample, the resin-impregnated fabric was pressed with a roller used in boat building, then wrapped with release film and pressed with non-stretch tape.

A rational method of reinforcing beams made of porous materials has been proposed. The experimental results of the proposed reinforcement indicate the high effectiveness of the proposed method of reinforcing porous materials.

All beams reinforced with carbon fabric were damaged due to reinforcement failure due to exceeding the permissible stress for pressure. Supporting the beams on supports with a larger surface area and a different shape could result in increased bending strength. Samples without reinforcement are characterized by a typical damage mechanism for bent beams.

Reinforcement and renovation of concrete structures using composite materials can become a technique for achieving structures’ required reinforcement and service life.

The technology can be used to significantly increase the load-bearing capacity of structures without increasing their weight and can insulate concrete, protecting it from corrosion [68].

In subsequent stages of the research, the authors will take into account the durability tests of the samples and will not conduct an industrial feasibility analysis.

## Figures and Tables

**Table 1:** Parameters of samples used in the preliminary and main experiments.

Sample	Material	Measuring [mm]	Unit of Mass [g]
AC1	aerated concrete, sample without additives	40 × 40 × 200	170
AC2	aerated concrete, sample impregnated with resin	40 × 40 × 200	182
AC3	aerated concrete, sample additionally reinforced with carbon fabric	40 × 40 × 200	190
AC4	aerated concrete, sample without additives	20 × 20 × 110	31
AC5	aerated concrete, sample impregnated with resin	20 × 20 × 110	36
AC6	aerated concrete, sample additionally reinforced with carbon fabric	20 × 20 × 110	41
VR1	volcanic rock, sample without additives	20 × 20 × 110	20
VR2	volcanic rock, sample impregnated with resin	20 × 20 × 110	26
VR3	volcanic rock, sample additionally reinforced with carbon fabric	20 × 20 × 110	33

**Table 2:** Results obtained in the preliminary experiment.

Sample	δ [MPa]	ε [%]	s [mm]	F_M_ [N]
AC1	1.84	84	0.7	784.8
AC2	2.75	108	0.9	1177.2
AC3	6.66	720	6.0	2882.9

**Table 3:** Results obtained in the main experiment.

Sample	δ [MPa]	Increase in Strength [%]	ε [%]	s [mm]	F_M_ [N]
AC4	1.69	base value	39	0.52	112.49
AC5	3.68	118	59	0.79	245.17
AC6	34.47	1940	749	9,99	2298.20
VR1	0.92	base value	54	0.72	61.24
VR2	2.27	147	80	1.07	157.10
VR3	25.14	2633	768	10.24	1675.73

## Data Availability

The original contributions presented in this study are included in the article. Further inquiries can be directed to the corresponding author.

## References

[1] Khayat K.H., De Schutter G. (2014). Mechanical Properties of Self-Compacting Concrete: State-of-the-Art Report of the RILEM Technical Committee 228-MPS on Mechanical Properties of Self-Compacting Concrete.

[2] Li N., Jin Z., Long G., Chen L., Fu Q., Yu Y., Zhang X., Xiong C. (2021). Impact Resistance of Steel Fiber-Reinforced Self-Compacting Concrete (SCC) at High Strain Rates. J. Build. Eng..

[3] Saif Y., Mallek J., Hadrich B., Daoud A. (2025). Mechanical Behavior of Self-Compacting Concrete Incorporating Rubber and Recycled Aggregates for Non-Structural Applications: Optimization Using Response Surface Methodology. Buildings.

[4] Zhang M., Chen J., Liu J., Yin H., Ma Y., Yang F. (2025). Fracture Behavior of Steel-Fiber-Reinforced High-Strength Self-Compacting Concrete: A Digital Image Correlation Analysis. Materials.

[5] La Scala A., Carnimeo L. (2025). Effective Comparison of Thermo-Mechanical Characteristics of Self-Compacting Concretes Through Machine Learning-Based Predictions. Fire.

[6] Stel’makh S.A., Shcherban’ E.M., Beskopylny A., Mailyan L.R., Meskhi B., Varavka V. (2022). Quantitative and Qualitative Aspects of Composite Action of Concrete and Dispersion-Reinforcing Fiber. Polymers.

[7] Mailyan L.R., Beskopylny A.N., Meskhi B., Shilov A.V., Stel’makh S.A., Shcherban’ E.M., Smolyanichenko A.S., El’shaeva D. (2021). Improving the Structural Characteristics of Heavy Concrete by Combined Disperse Reinforcement. Appl. Sci..

[8] Nikolenko S.D., Sazonova S.A., Asminin V.F., Mozgovoj N.V., Zvyagina L.N. (2022). Behavior of Dispersion-Reinforced Concrete under Dynamic Action. J. Phys. Conf. Ser..

[9] Kaddo M., Sinotova M. (2018). Study of Dry Mixes with Aluminate Cements for Self-Leveling Floors. IOP Conf. Ser. Mater. Sci. Eng..

[10] Kalkan Ş.O., Gündüz L. (2023). An Analysis of the Effectiveness of New Generation Self-Levelling Lightweight Composite Screed for Underfloor Heating Systems. J. Sustain. Constr. Mater. Technol..

[11] Zong M., Ma H., Yan X., Zhu P., Wang W., Liu H., Dong F., Hua M. (2025). Advances in the Application and Mechanism of Admixtures and Industrial By-Products in Cement-Based Self-Leveling Mortar: A Comprehensive Review. Materials.

[12] Lu J.-X. (2023). Recent Advances in High Strength Lightweight Concrete: From Development Strategies to Practical Applications. Constr. Build. Mater..

[13] Costa H., Carmo R.N.F., Júlio E. (2018). Influence of Lightweight Aggregates Concrete on the Bond Strength of Concrete-to-Concrete Interfaces. Constr. Build. Mater..

[14] Abhilasha K.R., Lakhani R., Mishra R.K., Khan S. (2023). Utilization of Solid Waste in the Production of Autoclaved Aerated Concrete and Their Effects on Its Physio-Mechanical and Microstructural Properties: Alternative Sources, Characterization, and Performance Insights. Int. J. Concr. Struct. Mater..

[15] Raj A., Borsaikia A.C., Dixit U.S. (2020). Evaluation of Mechanical Properties of Autoclaved Aerated Concrete (AAC) Block and Its Masonry. J. Inst. Eng. India Ser. A.

[16] Sidhu A.S., Siddique R. (2024). Review on Effect of Curing Methods on High Strength Concrete. Constr. Build. Mater..

[17] Paulík P. (2013). The Effect of Curing Conditions (In Situ vs. Laboratory) on Compressive Strength Development of High Strength Concrete. Procedia Eng..

[18] Pereira Prado L., Carrazedo R., Khalil El Debs M. (2022). Interface Strength of High-Strength Concrete to Ultra-High-Performance Concrete. Eng. Struct..

[19] Naganna S.R., Ibrahim H.A., Yap S.P., Tan C.G., Mo K.H., El-Shafie A. (2021). Insights into the Multifaceted Applications of Architectural Concrete: A State-of-the-Art Review. Arab. J. Sci. Eng..

[20] Ghalehnovi M., Asadi Shamsabadi E., Khodabakhshian A., Sourmeh F., De Brito J. (2019). Self-Compacting Architectural Concrete Production Using Red Mud. Constr. Build. Mater..

[21] Serralheiro M.I., De Brito J., Silva A. (2017). Methodology for Service Life Prediction of Architectural Concrete Facades. Constr. Build. Mater..

[22] Chang H., Wang P., Jin Z., Li G., Feng P., Ye S., Liu J. (2020). Durability and Aesthetics of Architectural Concrete under Chloride Attack or Carbonation. Materials.

[23] Barnat-Hunek D., Szafraniec M. (2021). Influence of Biodegradable Release Oils on the Physical and Mechanical Properties of Light-Colored Architectural Concrete. Materials.

[24] Tamayo-García B., Albareda-Valls A., Rivera-Rogel A., Cornado C. (2019). Mechanical Characterization of a New Architectural Concrete with Glass-Recycled Aggregate. Buildings.

[25] Subedi A., Kim H., Lee S.-J., Lee M.-S. (2025). Assessing Abrasion Resistance in Concrete Pavements: A Review. Appl. Sci..

[26] Plati C. (2019). Sustainability Factors in Pavement Materials, Design, and Preservation Strategies: A Literature Review. Constr. Build. Mater..

[27] Yang S., Yao X., Li J., Wang X., Zhang C., Wu S., Wang K., Wang W. (2021). Preparation and Properties of Ready-to-Use Low-Density Foamed Concrete Derived from Industrial Solid Wastes. Constr. Build. Mater..

[28] Cornaro C., Buratti C. (2020). Energy Efficiency in Buildings and Innovative Materials for Building Construction. Appl. Sci..

[29] Guo J., Huang M., Huang S., Wang S. (2019). An Experimental Study on Mechanical and Thermal Insulation Properties of Rubberized Concrete Including Its Microstructure. Appl. Sci..

[30] Celik S., Family R., Menguc M.P. (2016). Analysis of Perlite and Pumice Based Building Insulation Materials. J. Build. Eng..

[31] Song Q., Bao J., Xue S., Zhang P., Han X. (2022). Study on the Recycling of Ceramic Polishing Slag in Autoclaved Aerated Foam Concrete by Response Surface Methodology. J. Build. Eng..

[32] Chica L., Alzate A. (2019). Cellular Concrete Review: New Trends for Application in Construction. Constr. Build. Mater..

[33] Rudziewicz M., Maroszek M., Góra M., Dziura P., Mróz K., Hager I., Hebda M. (2023). Feasibility Review of Aerated Materials Application in 3D Concrete Printing. Materials.

[34] Bukhari S.A., Patil D., Gogate N.G., Minde P.R. (2023). Utilization of Waste Materials in Non-Autoclaved Aerated Concrete Blocks: State of Art Review. Mater. Today Proc..

[35] Thienel K.-C., Haller T., Beuntner N. (2020). Lightweight Concrete—From Basics to Innovations. Materials.

[36] Narayanan N., Ramamurthy K. (2000). Structure and Properties of Aerated Concrete: A Review. Cem. Concr. Compos..

[37] Cavalline T.L., Castrodale R.W., Freeman C., Wall J. (2017). Impact of Lightweight Aggregate on Concrete Thermal Properties. ACI Mater. J..

[38] Roberz F., Loonen R.C.G.M., Hoes P., Hensen J.L.M. (2017). Ultra-Lightweight Concrete: Energy and Comfort Performance Evaluation in Relation to Buildings with Low and High Thermal Mass. Energy Build..

[39] Samson G., Phelipot-Mardelé A., Lanos C. (2017). A Review of Thermomechanical Properties of Lightweight Concrete. Mag. Concr. Res..

[40] Fares H., Toutanji H., Pierce K., Noumowé A. (2015). Lightweight Self-Consolidating Concrete Exposed to Elevated Temperatures. J. Mater. Civ. Eng..

[41] Liu X., Chia K.S., Zhang M.-H. (2010). Development of Lightweight Concrete with High Resistance to Water and Chloride-Ion Penetration. Cem. Concr. Compos..

[42] Lotfy A., Hossain K.M.A., Lachemi M. (2016). Transport and Durability Properties of Self-Consolidating Concrete Using Three Types of Lightweight Aggregates. ACI Mater. J..

[43] Rashad A.M. (2021). An Overview of Pumice Stone as a Cementitious Material—The Best Manual for Civil Engineer. Silicon.

[44] Ma Z., Jiang L., Liao H., Cheng F. (2022). Research on the Methods for Improving the Compressive Strength of Solid Waste-Based High-Strength Autoclaved Aerated Concrete. Constr. Build. Mater..

[45] Onur Pehlivanlı Z., Uzun İ. (2022). Effect of Polypropylene Fiber Length on Mechanical and Thermal Properties of Autoclaved Aerated Concrete. Constr. Build. Mater..

[46] Peng Y., Liu Y., Zhan B., Xu G. (2021). Preparation of Autoclaved Aerated Concrete by Using Graphite Tailings as an Alternative Silica Source. Constr. Build. Mater..

[47] Cai Q., Ma B., Jiang J., Wang J., Shao Z., Hu Y., Qian B., Wang L. (2021). Utilization of Waste Red Gypsum in Autoclaved Aerated Concrete Preparation. Constr. Build. Mater..

[48] Liu H., Elchalakani M., Karrech A., Yehia S., Yang B. (2021). High Strength Flowable Lightweight Concrete Incorporating Low C3A Cement, Silica Fume, Stalite and Macro-Polyfelin Polymer Fibres. Constr. Build. Mater..

[49] Hosen M.A., Shammas M.I., Shill S.K., Jumaat M.Z., Alengaram U.J., Ahmmad R., Althoey F., Islam A.B.M.S., Lin Y. (2021). Investigation of Structural Characteristics of Palm Oil Clinker Based High-Strength Lightweight Concrete Comprising Steel Fibers. J. Mater. Res. Technol..

[50] Prakash R., Thenmozhi R., Raman S.N., Subramanian C., Divyah N. (2021). Mechanical Characterisation of Sustainable Fibre-Reinforced Lightweight Concrete Incorporating Waste Coconut Shell as Coarse Aggregate and Sisal Fibre. Int. J. Environ. Sci. Technol..

[51] Zeng Y., Tang A. (2021). Comparison of Effects of Basalt and Polyacrylonitrile Fibers on Toughness Behaviors of Lightweight Aggregate Concrete. Constr. Build. Mater..

[52] Wang C., Yin S., Zhao Y., Li Y. (2025). Flexural Behavior of Composite Beams with Textile Reinforced Concrete (TRC) Permanent Formwork Considering Interface Characteristics. J. Build. Eng..

[53] Karthikeyan G., Margret A.L., Vineeth V., Harshani R. (2023). Experimental Study on Mechanical Properties of Textile Reinforced Concrete (TRC). E3S Web Conf..

[54] Wu C., Pan Y., Yan L. (2023). Mechanical Properties and Durability of Textile Reinforced Concrete (TRC)—A Review. Polymers.

[55] Nahum L., Peled A., Gal E. (2020). The Flexural Performance of Structural Concrete Beams Reinforced with Carbon Textile Fabrics. Compos. Struct..

[56] Goliath K.B., Cardoso D.C., Silva F.D.A. (2021). Flexural Behavior of Carbon-Textile-Reinforced Concrete I-Section Beams. Compos. Struct..

[57] Raoof S.M., Bournas D.A. (2017). TRM versus FRP in Flexural Strengthening of RC Beams: Behaviour at High Temperatures. Constr. Build. Mater..

[58] Raoof S.M., Koutas L.N., Bournas D.A. (2017). Textile-Reinforced Mortar (TRM) versus Fibre-Reinforced Polymers (FRP) in Flexural Strengthening of RC Beams. Constr. Build. Mater..

[59] Witkowska-Dobrev J., Szlachetka O., Francke B., Chyliński F., Małek M., Šadzevičius R., Ramukevičius D., Frąk M., Dzięcioł J., Kruszewski M. (2023). Effect of Different Water-Cement Ratios on the Durability of Prefabricated Concrete Tanks Exposed to Acetic Acid Aggression. J. Build. Eng..

[60] Witkowska-Dobrev J., Szlachetka O., Dohojda M., Wiśniewski K. (2021). Effect of Acetic Acid on Compressive Strength and Geometric Texture of the Surface of C20/25 Class Concrete. Sustainability.

[61] Witkowska-Dobrev J., Szlachetka O., Malarski M., Czajkowska J., Miturski M., Nowak P., Dohojda M. (2023). Effect of Sewage on Compressive Strength and Geometric Texture of the Surface of Concrete Elements. Struct. Concr..

[62] Czajkowska J., Malarski M., Witkowska-Dobrev J., Dohojda M., Nowak P. (2021). Mechanical Performance of Concrete Exposed to Sewage—The Influence of Time and pH. Minerals.

[63] (2024). Eurocode 2: Design of Concrete Structures—Part 1-1: General Rules and Rules for Buildings.

[64] Merah A., Khenfer M.M., Korichi Y. (2015). The Effect of Industrial Coating Type Acrylic and Epoxy Resins on the Durability of Concrete Subjected to Accelerated Carbonation. J. Adhes. Sci. Technol..

[65] Coffetti D., Crotti E., Gazzaniga G., Gottardo R., Pastore T., Coppola L. (2021). Protection of Concrete Structures: Performance Analysis of Different Commercial Products and Systems. Materials.

[66] Markuszewski D. (2017). Detection and Tracking Damage in Composite Structures Elements. Mach. Dyn. Res..

[67] Markuszewski D. (2019). Comparison of Various Types of Damage Symptoms in the Task of Diagnostics Composite Profiles. Diagnostyka.

[68] Wiśniewski K., Dohojda M., Witkowska-Dobrev J. (2020). Ochrona Betonu Zwykłego Przed Agresywnym Środowiskiem w Budownictwie Rolniczym. Acta Sci. Pol.—Archit. Bud..

[69] Ajir K., Toufigh V., Ghaemian M. (2025). Protecting Ordinary Cement Concrete against Acidic and Alkaline Attacks Utilizing Epoxy Resin Coating. Constr. Build. Mater..

[70] Markuszewski D., Bielak M., Wądołowski M., Grzybek A. (2022). Polymer-Carbon Composite Supporting Structure. Adv. Sci. Technol. Res. J..

[71] Markuszewski D., Wądołowski M., Krajewski A. (2024). The Influence of Variable Stiffness of the Shape Memory Alloys Carbon Composite Structure on Mechanical Vibration. Materials.

[72] Gorzym M., Markuszewski D. (2024). Laboratory Tests of Rolling Resistance of Different Tread Profiles for the Wheel of Martian Roverr. Adv. Sci. Technol. Res. J..

[73] Wilczyńska K., Markuszewski D. (2024). Experimental Study of the Design of Strength Properties of Polymeric Support Structures. Chem. Rev..

[74] (2015). Specification for Masonry Units—Part 4: Autoclaved Aerated Concrete Masonry Units.

[75] (2019). Plastics—Determination of Flexural Properties.

[76] (2016). (Main) Methods of Testing Cement—Part 1: Determination of Strength.

[77] https://www.leica-microsystems.com/.

[78] Drobiec Ł., Niemiec R. (2023). Strength Parameters of Autoclaved Aerated Concrete. https://www.dnibetonu.com/wp-content/pdfs/2023/Drobiec_Niemiec.pdf.

[79] Kaminski M.L., Fasano E. Ultimate strength. Proceedings of the 14th International Ship and Offshore Structures Congress (ISSC).

[80] Boczkowska A., Krzesiński G. (2016). Kompozyty i Techniki Ich Wytwarzania.

